# Proposal for a Home Sleep Monitoring Platform Employing a Smart Glove

**DOI:** 10.3390/s21237976

**Published:** 2021-11-29

**Authors:** Remo Lazazzera, Pablo Laguna, Eduardo Gil, Guy Carrault

**Affiliations:** 1 Laboratoire Traitement du Signal et de l’Image (LTSI-Inserm UMR 1099), Université de Rennes 1, 35000 Rennes, France; remo.lazazzera@gmail.com; 2 Biomedical Signal Interpretation and Computational Simulation (BSICoS) Group, I3A, IIS Aragón, University of Zaragoza, and with the CIBER de Bioingeniería, Biomateriales y Nanomedicina (CIBER-BBN), 50018 Zaragoza, Spain; laguna@unizar.es (P.L.); edugilh@unizar.es (E.G.)

**Keywords:** sleep monitoring, apnea, heart rate variability, breathing rate, medical of things, smart glove

## Abstract

The present paper proposes the design of a sleep monitoring platform. It consists of an entire sleep monitoring system based on a smart glove sensor called UpNEA worn during the night for signals acquisition, a mobile application, and a remote server called AeneA for cloud computing. UpNEA acquires a 3-axis accelerometer signal, a photoplethysmography (PPG), and a peripheral oxygen saturation (SpO${}_{2}$) signal from the index finger. Overnight recordings are sent from the hardware to a mobile application and then transferred to AeneA. After cloud computing, the results are shown in a web application, accessible for the user and the clinician. The AeneA sleep monitoring activity performs different tasks: sleep stages classification and oxygen desaturation assessment; heart rate and respiration rate estimation; tachycardia, bradycardia, atrial fibrillation, and premature ventricular contraction detection; and apnea and hypopnea identification and classification. The PPG breathing rate estimation algorithm showed an absolute median error of 0.5 breaths per minute for the 32 s window and 0.2 for the 64 s window. The apnea and hypopnea detection algorithm showed an accuracy (Acc) of 75.1%, by windowing the PPG in one-minute segments. The classification task revealed 92.6% Acc in separating central from obstructive apnea, 83.7% in separating central apnea from central hypopnea and 82.7% in separating obstructive apnea from obstructive hypopnea. The novelty of the integrated algorithms and the top-notch cloud computing products deployed, encourage the production of the proposed solution for home sleep monitoring.

## 1. Introduction

Sleep is a physiological activity that influences daily life in different ways, and its measurement allows one to evaluate how well a person is sleeping. According to the U.S.A. National Sleep Foundation, sleep quality assesses if the sleep is restful and restorative [1]. It is fundamental for the health and well-being of people at all stages of life [2]. The U.S.A. National Institutes of Health (NIH) recognizes that chronic sleep deficiency and circadian disruption are emerging characteristics of modern urban lifestyles and are associated with public safety and increased disease risk, through multiple complex pathways in all age groups [3]. In children, sleep disordered breathing is associated with cardiovascular and metabolic risk factors, attention-related behavioral problems, and poor academic performance [4]. In the U.S.A., the Congress and the Department of Health and Human Services put sleep and circadian disturbances disorders as high priority targets for basic and clinical scientific investigation [4] to improve prevention, diagnosis, and treatment of such disturbances.

In the present paper, the authors attempted to answer two questions: which factors participate to sleep quality, and how sleep quality could be improved? Then, after a brief state-of-the-art introduction, a monitoring solution is proposed.

Sleep quality is mainly affected by a person’s habits and physio-pathological condition. Accordingly, monitoring vital signs during sleep can help to prevent, diagnose, and treat eventual sleep disorders.

The clinically acceptable sleep stages are majorly determined by reading the recorded EEG based on the R&K criteria. These criteria were standardized in 1968 by Rechtschaffen and Kales [5] and further developed by the American Academy of Sleep Medicine in 2007 (AASM 2007) [6]. Various features of the electroencephalogram (EEG) signals have been proposed to study the sleep dynamics, among those are time-domain summary statistics, spectral analysis, coherence, time-frequency analysis, and entropy, to name a few [7,8,9,10].

Polysomnography (PSG) is the gold standard procedure used to identify any sleep disorder. It consists of an overnight recording of different electrophysiological signals such as EEG, electromyogram, electrooculogram, electrocardiogram (ECG), airflow, peripheral capillary oxygen saturation (SpO${}_{2}$), and photoplethysmogram (PPG). The acquisition and analysis of these signals require human expertise and specialized equipment; for this reason, it is mostly performed in medical clinics. As it is a very uncomfortable and costly procedure, sleep disorders are often underdiagnosed.

Polygraphy, instead, is a less restrictive examination conducted at home, for the simplest cases to diagnose. Usually, it requires the use of a nasal cannula to detect the nasal pressure airflow, which is not comfortable to wear and, in many cases, interferes with natural sleep. However, it does not record EEG and thus is not useful for sleep staging.

In the present manuscript, the authors proposed a novel sleep monitoring platform based on a smart glove device called UpNEA, a smartphone application, and a remote server called AeneA. The system intends to propose a less invasive alternative to the PSG and polygraphy sleep monitoring solutions. The solution aims to keep track of two main vital parameters: respiration and heartbeat using PPG and SpO${}_{2}$ sensors. In addition, the proposed system integrates some already validated algorithms for detecting abnormalities in the heart rate and breathing rhythm.

The choice of surveilling only these two physiological variables is dictated by the less incommodious equipment needed to record such signals, and the research effort is focused on attempting to retrieve as much information as possible from the acquired records to compensate for the lack of other PSG sensors.

Breathing rate (BR) is an important physiological indicator to diagnose a variety of chronic diseases such as pneumonia and, eventually, cardiac arrest [11]. The gold standard measurement technique for BR is capnography, but it requires cumbersome equipment that is uncomfortable for long-term monitoring. To reduce the discomfort, recent publications have explored the way of deriving the respiration signal from the electrocardiogram (ECG) and PPG signals [12,13,14]. Respiration activity modulates both ECG and PPG signals [15]. The heart rate increases during inspiration and decreases during expiration: this phenomenon is known as respiratory sinus arrhythmia [16,17]. The decreased stroke volume in cardiac output, due to ventricular filling, causes respiration-induced amplitude variation in the PPG signal [18,19]. Indirect estimation of BR estimation is constituted by amplitude, frequency, and baseline wander modulations of ECG and PPG signals [13,20,21].

A respiratory sleep disorder that is worth monitoring is sleep apnea and hypopnea. Sleep apnea is the cessation of breathing during sleep, while hypopnea is characterized by abnormal reductions of the respiratory flow [22]. Both can be obstructive or central: obstructive if the breathing effort continues while there is a mechanical obstruction of the airways (resulting in interruption of the airflow); central if no breathing effort nor airflow is present. Sleep disordered breathing affects more than 15% of the U.S.A. population and causes daytime sleepiness, injuries, hypertension, cognitive impairment, risk of heart attack, stroke, and mortality [4,23,24,25]. The golden standard apparatus to diagnose such disturbances is either PSG or polygraphy, but nowadays different noninvasive techniques have been extensively developed [26].

During sleep, cardiac arrhythmia could even represent a medical emergency for individuals with cardiovascular diseases [27]. In these cases, heart rate (HR) long-term and continuous monitoring are beneficial for a sudden intervention.

Bradycardia and tachycardia are HR abnormalities defined as excessively decreased or elevated rates of the heart. Such conditions can occur in specific situations such as during sleep, relaxation, or physical activity [28]. Generally, an HR < 50 beats per minute (bpm) can cause fatigue and transient dizziness. Tachycardia is present when an individual presents an HR higher than 120 bpm: this can lead to palpitations, shortness of breath, or dyspnea on exertion [29].

Atrial fibrillation (AF) is predominantly associated with an increased risk for heart failure and stroke. Its prevalence increases with age [30,31,32], and its nature can be intermittent and asymptomatic [33]. Although is the most common arrhythmia, for a large part of the U.S.A. population it is undiagnosed [34]. Instead, premature ventricular contraction (PVC) occurs when an ectopic focus, originating in the ventricles, leads to premature activation of the ventricles, antecedent to typical sinoatrial node activation [35]. PVCs can cause symptoms as the sensation of an irregular pulse or having skipped beats, but concerning AF, the clinical course of patients with PVCs is typically benign [36].

To investigate the presence of HR abnormalities, the golden standard methods are the 24-h Holter monitor or the 30-day event recorder [37], employing ECG electrodes adhered to the person’s chest. In recent years, a wearable solution for long-term HR monitoring implements wrist-wearable PPG sensors to continuously measure HR in free-living conditions [38]. The pulse rate time series, derived from PPG, is a surrogate of the heart rate time series and so the pulse rate variability (PRV) concerning the heart rate variability (HRV), as demonstrated in [39] during tilt table tests. HRV represents fluctuations in the heart rate related to autonomic nervous system control: HRV high-frequency components (between 0.15 and 0.4 Hz) represent the vagal tone, while low-frequency (LF) components (from 0.04 to 0.15 Hz) manifest the activation of both parasympathetic and sympathetic nervous systems. Both the PRV and the ratio between LF and HF (defined as the sympathovagal balance [40]) have been used to discriminate sleep breathing disorders [41,42].

### State-Of-The-Art

Nowadays, different devices have been proposed for sleep monitoring solutions employing less invasive technology, attempting to shift the sleep observation task from medical clinics to home health care. The main objectives of this transition consist in improving the quality of records by increasing user comfort during sleep monitoring and making sleep diagnosis more accessible.

For example, the Advanced Brain Monitoring Company developed the Sleep Profiler PSG2 EEG measuring helmet that employs also a nasal cannula: although the proposed equipment is reduced in size concerning the PSG one, it has the benefit of diagnosing sleep disorders with the same accuracy but, still, the level of discomfort on the user is not reduced. Similar commercial equipment for sleep home diagnosis exists on the market, e.g., the Edentec Monitoring System, Embletta, SOMNOcheck, and Apnea Risk Evaluation System (ARES). All of those implement a helmet as well as an airflow nasal cannula to keep track of respiration, increasing the accuracy of BR estimation, yet making the equipment cumbersome. Some other, like Sibel Home, ApneaLink, and Stardust II, implement chest belt and limb sensors to increase the user’s comfort and derive physiological signals from pressure sensors, PPG, and accelerometers: this solution reduces monitoring accuracy and still needs physical connections that can be easily dislodged during sleep [43,44,45].

To cite other cases, the Stadius Center laboratory at KU Leuven is also working on a bed-integrated platform that aims to diagnose and treat apneas, implementing plethysmography, respiration, and ballistocardiography signals. In the literature, Hernandez et al. [46] proposed a sleep monitoring device called PASITHEA, tailored for apnea detection: the device exploits a nasal cannula to detect the airflow interruption and then acts with a kinesthetic actuator for vagal stimulation.

Some other approaches aimed to reduce the complexity of the PSG system, making use of ECG signals to extract vital parameters as HR and BR [47] and then differentiate between obstructive and central apnea events [48,49]. Some others also integrated accelerometer signals besides ECG to seek better accuracy [50,51].

Some studies evaluated the performance of an unobtrusive sleep monitoring system by operating a pressure sensor into a bed mattress [52] for the identification of the sleep apnea–hypopnea syndrome. In [53], instead, the sensor incorporates multiple pressure transducers to surveil respiration, heart rate, and body movements on sleeping subjects. Results of this study were promising to diagnose sleep apnea–hypopnea but are not yet sufficient to exploit the solution for medical purposes.

In recent years, several studies explored the opportunity to exploit smartphones as sleep monitoring devices, indicating encouraging results. Many works employed smartphone-based accelerometry for respiratory rate assessment [54,55,56] and so to assess sleep quality [57]. In particular, in a proof-of-concept study [58] tested the feasibility of using smartphone accelerometry to characterize the sleep respiratory activity and identify obstructive sleep apnea events. Further [59,60], full-night audio signals recorded with a smartphone microphone were provided to an automatic detector for identifying sleep apneas and hypopneas. Nevertheless, the pilot studies have not yet passed medical certification.

A project similar to the sleep monitoring platform presented in this work is being developed by EIT Health and consists of a smart strap called ApneaBand, that records PPG signals on the wrist. The signal processing algorithms are embedded in the device, and thereby they do not take the computational advantage of cloud computing and they need to be updated over the air. To the authors’ knowledge, PPG signals acquired at the level of the wrist would be more affected by noise than those collected on the index finger, and this could reflect on the sleep monitoring performances. Besides, the ApneaBand results are not immediately available to the user after sleep as the device needs to be connected to a personal computer for data visualization.

A second interesting commercial device, very close to the monitoring system proposed in this paper, is WatchPAt by Itamar Medical™, that implements the same sensors UpNEA is equipped with. WatchPAt integrates the patented Peripheral Arterial Tonometry (PAT${}^{\text{®}}$) technology to track sleep and detect central sleep apnea events. Concerning this solution, whose architectural design has not been revealed for the company interests, the sleep monitoring platform presented in this paper adds more functionalities to track sleep with, e.g., the possibility to classify apnea and hypopnea events and even discern if their nature is central or obstructive.

In this context of a variety of sleep monitoring solutions, the system proposed in the present manuscript aims to address the problem of increasing detection accuracy of sleep disturbances, by implementing sensors that are less cumbersome concerning those employed in PSG.

The authors, having already published a method to detect and classify sleep apnea/hypopnea syndrome from PPG signals [42] and having compared the performances of different algorithms for BR estimation in [61], wanted to concertize those works in a novel integrated platform for sleep monitoring. The proposed system has been built employing the most recent hardware material and one of the top-notch cloud computing products. The whole architecture based on the cloud offers a high computational workload and scalability and easily allows the integration of new algorithms and connected devices.

The novel platform consists of a connected glove called UpNEA, a smartphone application, and a Matlab Production Server™ called AeneA hosted by Amazon Web Services (AWS). To the authors’ knowledge, there is no similar publication in the literature about a sleep monitoring system employing such technology. We propose that the present publication could inspire the development of new Medical of Things devices, coordinated by a cloud computing ecosystem.

## 2. Materials and Methods

The proposed platform works as follows. At first, the smart-glove UpNEA collects PPG, SpO${}_{2}$, and 3-axis accelerometer signals. Those signals are encoded by the device hardware and then sent via Bluetooth Low Energy (BLE) to a mobile application. The mobile application sends these data to a secure remote server (AeneA) for cloud computing. The server decodes the received data and reconstructs the overnight signals. Then, data are analyzed by different algorithms to estimate the overnight states of sleep, breathing, and heart rate. Afterward, detection methods are deployed to observe and classify apneas and hypopneas, and episodes of tachycardia, bradycardia, atrial fibrillation, and premature ventricular contraction. Finally, the analysis results are then stored in a SQL database and accessed by a smartphone or web application to show the overnight report. The web application is conceived to be available for the user as well as for the user’s clinician. The purpose of the proposed system is to make the user aware of potential sleep disorders and ultimately to monitor the effects of the person’s habits on sleep. The purpose of such a platform would be to make a first home screening night sleep possible, instead of going to specialized clinics. As an effect, it would reduce the sleep diagnosis costs and make the task more comfortable.

In the following subsections, the proposed novel platform is described by detailing its components and their functionalities. The integrated algorithms performances have been recalled in the results section and, as a proof-of-concept, the proposed platform has been tested during four overnight recordings on a healthy subject.

### 2.1. Data Acquisition and Cloud Computing

**Figure 1:**
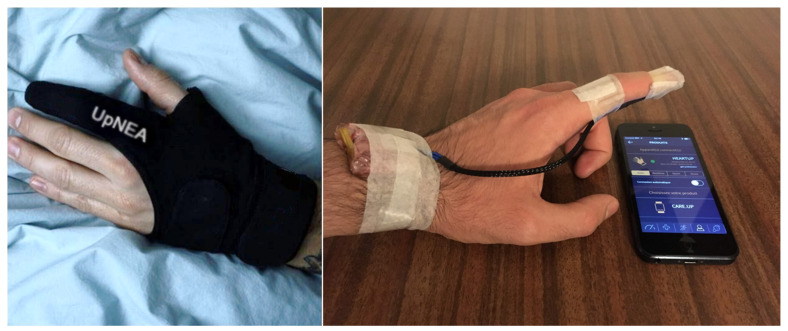
The UpNEA device and its prototype.

The smart glove UpNEA and its hardware prototype are both shown in Figure 1. The device has a 32-bit PIC32MX340F512H processor unit (MicroChip${}^{\text{®}}$, Chandler, AZ, USA). It embeds two reflected mode PPG MAX30101 sensors (Maxim Integrated™, San Jose, CA, USA), one using red light and one using infrared frequencies, an internal RAM of 32KB, an external flash memory of 512KB, and a MAX21105 inertial measurement unit (Maxim Integrated™, San Jose, CA, USA). The power system consists of a lithium battery packaged close to the electronic custom board. The connected glove is controlled by a mobile application that allows the device to start recording signals, receive data, and terminate the acquisition.

The PPG signals are sampled at 100 Hz and 3-axis accelerometer data are sampled at 50 Hz. The SpO${}_{2}$ value is computed in a temporal window of a time pulse by taking as input the two PPG signals and resampling at 1 Hz using an embedded algorithm in the microprocessor. For cloud computing, only one of the two PPG signals was considered: the one operating in the red light frequency.

**Figure 2:**
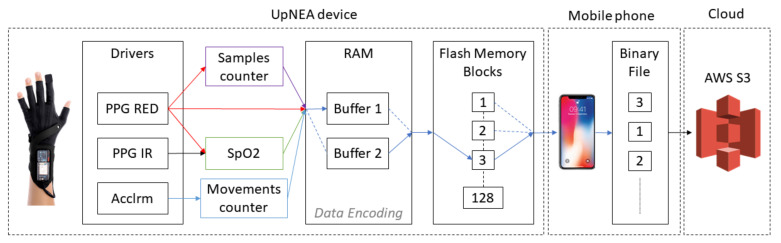
Datastream visualization for UpNEA.

The data acquisition process is visualized in Figure 2. As the acquisition starts, data are stored in the UpNEA internal memory and then transferred to the external flash memory. The external flash memory of UpNEA is organized in blocks of 4 KB, and for this reason, the internal RAM allocated for this task consists of two buffers of 4 KB each.

At the beginning of acquisition, the first buffer is filled with the incoming encoded data, and when it is full, the second buffer starts to fulfill. As the second buffer is used for storing data, a parallel process moves data from the first buffer to the external flash memory. Then, when the second buffer is full, this process is repeated by re-allocating data in the first buffer and moving data from the second. This process is repeated until the acquisition ends.

Once a data block of 4 KB has been recorded on the external flash memory, the UpNEA device looks for an available BLE communication with the smartphone. If the communication is available, the block is sent by BLE to the mobile application on the smartphone. This block is sent in an object containing the binary data of the acquisition and also a JSON header, in which are specified the device ID and a checksum performed with XOR operator on 8 bits. During the BLE transmission, the UpNEA device still acquires and stores new data from the pulse oximeters drivers.

The task performed by the mobile application consists of collecting all the blocks sent by the smart glove, verifying the checksum, and concatenating all the blocks in a binary (.bin) file allocated in the smartphone memory. If the checksum is not verified, the mobile application sends to UpNEA a resend request for the specific block of data; otherwise, the external flash memory block of the device is freed. Doing so, it is worth noticing that the arrangement of the blocks in the binary file is not chronologically ordered because it depends on the blocks’ order of the UpNEA external flash memory. Additionally, in order to univocally associate each sleep session event to the current user account profile, the filename of the binary file consists of the user-ID and time information.

Finally, when the user wakes up, they send a STOP message to UpNEA by using the mobile application on the smartphone. This immediately aborts the recording data process and finalizes the data transmission. Once all the data from UpNEA are correctly received by the mobile application, the binary file is uploaded to a data bucket AWS S3, where the information is stored.

When a new file in the data bucket is stored, an AWS Lambda function is triggered. This function receives as input the filename of the new binary file and establishes, via IP, a connection with Matlab Production Server™ (MPS), hosted in a m5.xlarge AWS Elastic Computing (EC2) instance. Subsequently, the AWS Lambda sends an HTTP request to MPS, calling a specific MATLAB${}^{\text{®}}$ deployed function (passing as input the filename), which in turn starts a connection request with AWS S3 to download the specific binary file. Once the binary file has been locally downloaded on the EC2 instance hard drive, the MATLAB${}^{\text{®}}$ deployed function reconstructs the signals by decoding the received data. Afterward, different signal processing algorithms are applied to the acquired data for

- heart rate estimation,
- tachycardia and bradycardia detection,
- atrial fibrillation and premature ventricular contraction detection,
- breathing rate estimation,
- apnea and hypopnea detection and classification, and
- sleep stages classification.

**Figure 3:**
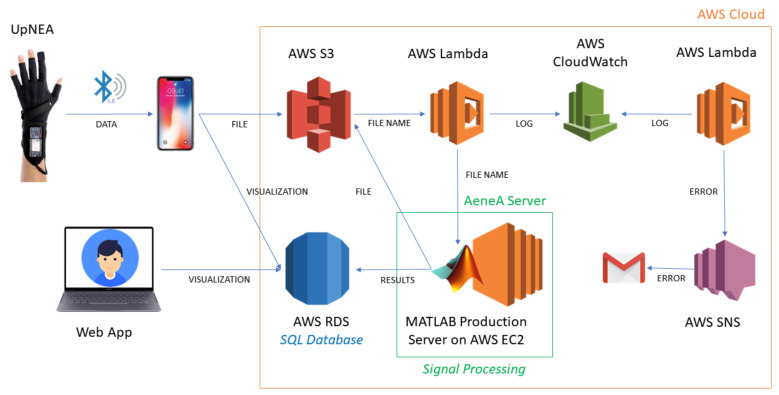
UpNEA AWS architecture for cloud stream and computing, refer to text for further details.

The whole cloud computing system is represented in Figure 3, and the elapsed time for computing all these tasks is ~30 s. The AWS platform was chosen for its scalability (capacity to easily upgrade its hardware characteristics) and robustness to handle data stream and perform cloud computing. The chosen EC2 hardware consists of four 3.1 GHz Intel Xeon Platinum processors, 16 Gb of RAM memory, 50 GB storage memory EBS-Only, network bandwidth up to 10 Gbps, and EBS bandwidth up to 3500 Mbps.

After processing, all the results labeled with the user-ID and sleep session time are recorded in an AWS Relational Database Service (RDS) for Microsoft SQL database, with one-minute resolution: the heart rate and breathing rate are averaged.

Finally, the user can visualize the overnight report just 45 s after waking up, by using the mobile or web application hosted by EC2. The application reads the SQL database and displays the overnight report containing the plots of the extracted information.

To handle the errors, a web service AWS Cloudwatch has been deployed. Any error generated by the MATLAB${}^{\text{®}}$ deployed function, or by the AWS system, is communicated to an AWS Lambda that writes the error in the log file of AWS Cloudwatch. Errors in the log trigger a second AWS Lambda function that calls the AWS Simple Notification Service (SNS) to notify the web administrator by a report email.

### 2.2. Data Compression and Encoding

**Figure 4:**
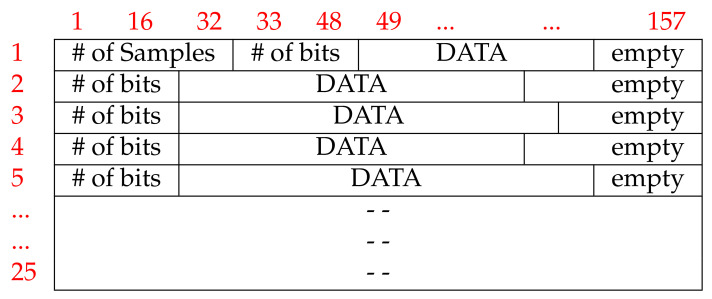
Encoding block structure.

UpNEA sends objects containing a JSON header and a binary block of 4KB to the smartphone application via BLE. However, before being recorded on hardware memory, data are compressed and encoded. Each binary block is composed of 25 rows of 157 Bytes. The first row of each block contains four elements: the number of samples, the number of useful bits, the encoded data, and, eventually, empty bits. The following 24 rows of each block, present all the same structure of the first one, except for the first element, see Figure 4.

The first element of the block indicates the number of samples produced by the drivers from the beginning of the acquisition up to the first PPG sample, included in the block. This element is stored in 4 Bytes LSBFirst; knowing that the sampling frequency of the PPG drivers is 100 Hz, allows reconstructing the time sequence. The time instants associated with a SpO${}_{2}$ value or to a sleep movement correspond to those of the last PPG sample, stored in the block.

The second element of the first block row is a 2 Bytes element LSBFirst, containing the number of useful bits in that row. This number can be smaller than the remaining row capacity; in this case, the last bits contain no information.

The encoded data are the next element present in the row of the block and, eventually, are followed by empty bits. Due to the encoding method, it was not possible to ensure the same amount of information bits in each row of the block. In Figure 4, the block structure is visually represented. The compression method of a data element in each block row is lossless and the encoding is based on empirical Huffman encoding [62]: a prefix (data descriptor) has been assigned to each element of the data stream, allocating less memory for the most frequent value. The first data value per block is always the absolute value, (*AV*) of the PPG signal, encoded in 18 bits. Because of the higher sampling rate of PPG, the next samples of PPG are encoded as Delta Differences (*DD*) between the new sample and the old one. The *DD* prefix is encoded with the data descriptor *0* and the *DD* values are stored in *signed int* of 8 bits. If *DD* requires more bits to be stored, then the new PPG sample value is encoded as the *DD* value, devised by a Scale Factor (*SF*). The data stream, then, includes the *SF* descriptor, the *SF* value, and the *DD* value. The *SF* is encoded on 3 bits, so it can encode 8 values. In the case that the *SF* is too high to be encoded as *unsigned int* on 3 bits, that sample is assumed to be an artifact, so it is coded as Garbage (*GB*) without recording its value but always incrementing the sample counter by one unit. After *GB* data, an *AV* descriptor and its value are proposed to restore the encoding and compression.

The number of sleep movements is encoded on 16 bits, while the SpO${}_{2}$ values on 6 bits. This allows the SpO${}_{2}$ to have values in the range [0, 63]. For this reason, when a SpO${}_{2}$ is produced, it is decreased by 37 and the result is stored in the block. If the difference result is negative, it is replaced by 0 as evidence of an artifact in the signal. The data descriptor’s size is variable, and it has been chosen in order to assign to the most frequent value, the lowest memory allocation. The data descriptors and data memory allocations are listed in Table 1, and an example of an encoding line is presented in Table 2.

Data decoding and decompression are performed by the MATLAB${}^{\text{®}}$ deployed function in MPS. This function reads the binary file stored in AWS S3, per 4KB blocks. The blocks are then chronologically ordered and the signals are reconstructed by decoding each row of the block. If a block is corrupted or GB segments are present, then the signal segment is discarded from the analysis.

### 2.3. PPG Signal Processing

#### 2.3.1. Heart Rate

At first, the PPG signal is filtered by removing its mean and dividing it by its standard deviation (z-score normalization). Each peak of the PPG signal represents the pulse occurrence time, and to get them, a search rule is used. It detects the peaks with a minimum prominence, fixed by empirical analysis on the database. The peak prominence is an adimensional parameter and depends on the representation of the acquired PPG signal by the acquisition device. It measures how much the peak stands out due to its intrinsic height and its location, relative to other peaks. In this work, for the peaks search implementation, the MATLAB${}^{\text{®}}$ signal processing toolbox is used [63]. This strategy was chosen to discriminate among pulses, discarding some pronounced dicrotic notch of the PPG signal that otherwise could be detected as single pulses.

To detect episodes of bradycardia and tachycardia, the method developed by Bonomi et al. in [64] was implemented. The beat time duration has been computed as the time difference between two successive pulses. To discover and handle ectopic pulses, the method in [65,66] was implemented, under the assumption that the pulse rate time series is a surrogate of the heart rate time series. The estimated pulse rate was used to determine the average inter-pulse interval (IPI) in one minute window. The IPI indicates the mean time between pulses, and it was used as the surrogate value for inter-beat intervals (IBI), extracted from ECG. Bradycardia was defined, in a one-minute window, as any episode during which the IBI was >1200 ms (HR < 50 bpm); tachycardia, instead, as any episode during which the IBI did not exceed 500 ms (HR > 120 bpm).

AF and PVC are detected implementing the method developed by Rademeyer [67] and embedded in a wireless device to monitor psychiatric patients. The Rademeyer algorithm receives as input the electrocardiogram R-R time interval series, and in the present work, they have been replaced by the pulse-to-pulse time instants (under the assumption that the R-R time series is a surrogate of the pulse rate time series [39]). Primarily, an average threshold is defined as the average of R-R time intervals in the series. Then, a possible AF is recognized when a pulse-to-pulse interval changes suddenly by more than 50 ms above the average threshold [68]. When the HRV curve drops below the average threshold by more than 50 ms (because the next pulse takes place later), a possible PVC is detected. Finally, each one-minute segment of the night recording is labeled as AF or PVC according to the presence of at least one of these detections.

#### 2.3.2. Breathing Rate

The embedded algorithm for the breathing rate estimation is the one implemented by Khreis et al. in [14].

The choice of the best respiration rate estimation method was performed by the authors in a comparative study published in [61]. For comparison, several techniques were implemented: empirical mode decomposition (EMD), EMD combined with principal component analysis, wavelets analysis, respiratory-induced intensity variation analysis, respiratory-induced amplitude variation analysis, respiratory-induced frequency variation analysis, and Kalman Smoother method from data fusion. The algorithms were validated on the public CAPNOBASE database [69], which consists of 42 subjects (29 children and 13 adults) with simultaneous ECG and PPG recordings. Eight minutes of high-quality data have been acquired during elective surgery or routine anesthesia for each subject. The capnography waveforms were manually labeled by the authors of the study, and the annotations of BR were used as reference values; methods comparison was performed by segmenting the PPG signal in windows of both 32 s and 64 s.

Finally, the best performing algorithm was one implemented by Khreis et al. in [14], that tracks the respiration signal, using the Kalman smoother, to fuse modulation signals with the highest respiratory quality indices.

#### 2.3.3. Apnea and Hypopnea

Authors developed and published new methods for sleep apnea and hypopnea detection and classification. Those algorithms were integrated into the proposed platform for cloud computing their complete description and validation are presented [42].

**Figure 5:**
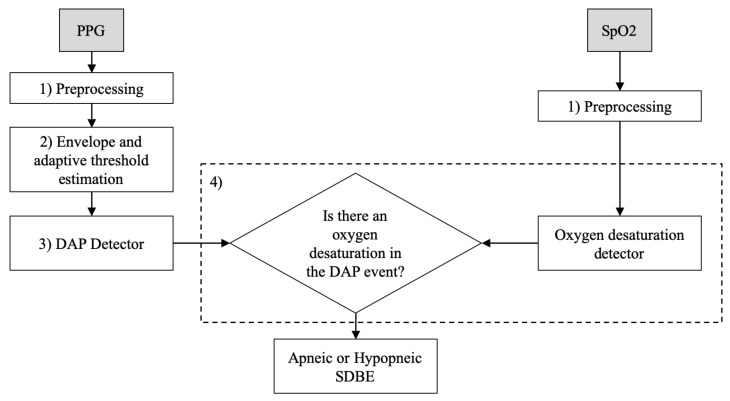
Apneic and hypopneic events detection flowchart.

Briefly, the underlying principles of the detection and classification methods reside in a decrease in amplitude fluctuation (DAP) of PPG signal, usually linked to a sleep disordered breathing event, as shown in [70,71,72,73,74,75]. From this perspective, the embedded apnea/hypopnea detection method implemented a DAP detector. A DAP event was considered apneic/hypopneic when oxygen desaturation also occurred in a 20 s temporal window left-centered on the DAP. The detection flowchart is shortly described in Figure 5.

To further differentiate between sleep disordered breathing events types, PRV-based features were extracted from 65 s PPG temporal windows, centered on the DAP, in order to account for sympathovagal arousals. Ultimately, the classification algorithm used a Fine Gaussian Support Vector Machines (SVM) classifier, with Gaussian kernel with 0.56 scale, to differentiate between apneic or hypopneic, obstructive or central DAP events.

The methods were tested on 96 overnight signals, recorded at the UZ Leuven hospital, from patients with sleep apnea/hypopnea syndrome and without any kind of collateral morbidity. Each record contained a PPG and a SpO${}_{2}$ signal sampled at 500 Hz, and the apnea–hypopnea index (AHI) was calculated as the number of respiratory events per hour of sleep, scored according to the AASM 2012 rules [76]. The average AHI in the UZ Leuven hospital dataset is 31.3 and 39% of the subjects had an AHI larger than 30; 53% of them had an AHI between 5 and 30 and the remaining 8%, an AHI less or equal to 5. The annotations contain the beginning and duration of central apnea (CA), central Hypopnea (CH), obstructive apnea (OA), and obstructive hypopnea (OH). Table 3 shows the total number of annotations per sleep disruptive breathing event (SDBE) category, in the database.

For the overnight sleep apnea/hypopnea detection, each PPG recording was divided into segments of one minute. Then, 3-fold cross-validation (CV) was performed as follows: at each fold, two-thirds of the patients present in the database were used to train the detectors and one-third to test the trained detectors. The 3-fold CV was performed per patient without re-substitution. As PPG features can be patient-specific, it was taken into account that each patient should be either in the detection test set or in the detection training set, in order to ensure a good generalization on new subjects.

Concerning the SDBE classification in CA, OA, CH, and OH, a total of 37 features were extracted from both the PPG and the SpO${}_{2}$ signals corresponding to a time frame containing the SDBE. A dataset containing labeled extracted features was then built up regardless of the implementation of a Leave One Subject Out (LOSO) procedure. That choice was taken because of the heterogeneous distribution of SDBE types among patients: for this reason, a further step consisted of balancing data so that each class would be represented by the same amount of information. Features selection was then applied to reducing the dimensionality of the problem. The Fine Gaussian Support Vector Machine model was then trained and tested by using 10-fold CV.

#### 2.3.4. Sleep Stages

A movements counter has been used to classify sleep stages. The counter is based on the 3-axis accelerometer signal, captured by the UpNEA device. Deep Sleep (*DS*) is defined as a stage when no movements or poor movements are detected, Light Sleep (*LS*) when frequent little movements are recorded, and Awake (*AW*) when a huge number of movements are detected. In the present subsection, the night movement detection and the sleep stages classification are described.

The accelerometer sampling rate is 50 Hz, therefore every 20 ms (δ) the device acquires the 3-axes accelerometer values $x_{i},\;y_{i},\;z_{i}$. These values are compared with the previous ones $x_{i-1},\;y_{i-1},\;z_{i-1}$ by computing a derivative $D_{i}$:

(1) $$D_{i}=\frac{\left|x_{i}-x_{i-1}\right|\;+\;\left|y_{i}-y_{i-1}\right|\;+\;\left|z_{i}-z_{i-1}\right|}{\delta }$$

If $D_{i}$ is larger than a certain threshold, then a movement has been detected. The value for the threshold is empirically chosen and depends on the hardware characteristics.

The total number of movements at each *j*-th minute ($N^{j}$) is then collected. When the overnight recording is terminated by the user, the night movements mean ($m_{N}$) and its standard deviation ($\sigma_{N}$) are computed. A sleep stage label is then assigned to each night minute, following these criteria:

(2) $$\begin{array}{c} if\;(N^{j}>m_{N}+3\times \sigma_{N}):j\in AW;\\ else\;if\;(N^{j}>m_{N}+\sigma_{N}):j\in LS;\\ otherwise\;j\in DS. \end{array}$$

If the number of movements performed by the user overnight is greater than the night movements mean plus three times its standard deviation, then we say that likely the user was awake; otherwise, two more scenarios are possible to light or deep sleep.

### 2.4. SQL Database

Once AeneA completes the cloud computing analysis, the MATLAB${}^{\text{®}}$ deployed function writes all the information extracted from signals, in the SQL database. Two types of data are recorded: statistical overnight data and one-minute overnight data. Statistical overnight data comprehend all the statistics computed on the overall sleep duration, whereas one-minute overnight data collect all one-minute overnight information. These two types of data are stored in two different database tables, called, respectively, *NightSleepSummary*, for statistical overnight data, and *SleepEvents* for one-minute overnight data. Each overnight UpNEA record adds one line to the table *NightSleepSummary* and as many lines as the number of sleep minutes to the table *SleepEvents*. Table 4 visualizes the two SQL tables fields, corresponding to the data input in the database. The *NightSleepSummary* presents fields containing the counters for apneas, PVC and AF, tachycardia, and bradycardia events, as well as the times the user woke up during the night. Moreover, in the same table are also recorded the overnight mean HR, BR, and SpO${}_{2}$ values, with user information and the sleep stages total duration. In *SleepEvents* table, instead, are reported the user information per sleep night minute: these data collect the average HR, BR, and SpO${}_{2}$ estimates within the minute, adding also the eventual presence of arrhythmia and apneas.

### 2.5. UpNEA Web Application

**Figure 6:**
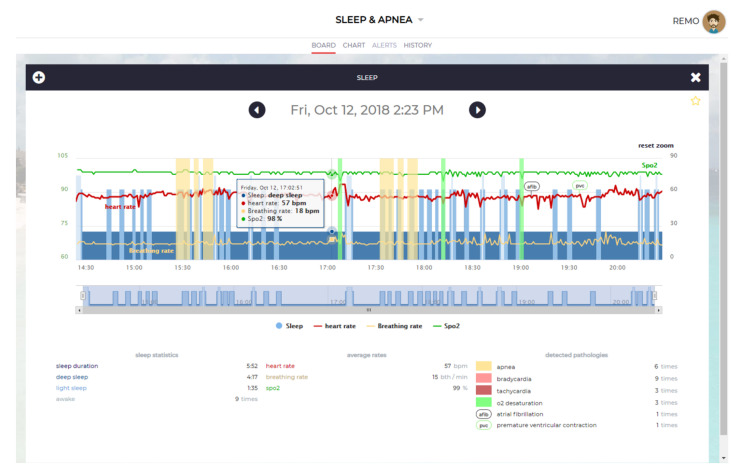
The user interface of the UpNEA web application.

The user can access their overnight data by logging in to a dedicated internet web page hosted by AeneA, or in a smartphone application. The user can navigate through the recorded nights and select which one to display. The plot implements the Highcharts JavaScript charting engine from Highsoft, to represent the overnight physiological signals. The charting engine imports data from the SQL database and displays them on a web page. Figure 6 presents the screenshot of a generic overnight plot, without clinical significance. The chart is composed of a zoomable time series with a range selector bar at the bottom, followed by a legend. The range selector displays only the overnight sleep stages, and a legend is an active tool that allows showing/hiding information on the plot.

Signals are extracted from the *SleepEvents* database table, with apnea, AF, PVC, tachycardia, and bradycardia detections. Statistical data, instead, are extracted from the *NightSleepSummary* database table. The plot shows HR, SpO${}_{2}$, and BR signals; apnea and hypopnea detections; as well as the tachycardia and bradycardia events, represented by colored bands. In the background, sleep stages are represented in different blue tonalities. To not overcharge the visualization, the red bands of tachycardia and bradycardia have been deselected from the figure. Each sleep disruptive breathing event detection is labeled as apneic or hypopneic, central or obstructive when the mouse pointer passes over the yellow bands. Ultimately, overnight statistics are reported below the graphic.

## 3. Results

In the present section, the performances of the methods embedded in the proposed platform for sleep monitoring will be recalled. Then, a preliminary study employing the novel sleep monitoring system and consisting of four overnight recordings, on a healthy subject, will be presented.

### 3.1. Performances of Methods Deployed in the Novel Sleep Monitoring Platform

At first, the efficacy of the data compression and encoding algorithms will be presented, then the performances of the breathing rate estimation methods followed by those obtained from the apnea and hypopnea detection and classification analysis. Concerning the performances of the pulse rate estimation method and those of the AFib and PVC detection, the authors give the reader the possibility to seek further information by approaching the original manuscripts. At last, during the testing phase, it has not been possible to evaluate the sleeping stage classifier based on movement detection; however, the authors decided to integrate it into the platform as an indicative prediction index.

#### 3.1.1. Data Compression and Encoding

The compression algorithm used to encode the data is lossless and acts also as a noise detector when the difference in amplitude between two adjacent PPG samples (sampled at 100 Hz) cannot be allocated in an 8-bit integer. The average binary file size produced during one sleep session of eight hours is approximately 5 Megabytes. Reduced file size has two impacts on the proposed sleep monitoring architecture: it lowers both the time spent by the smartphone to transmit data into the AWS S3 bucket and the transfer time between UpNEA and the smartphone, and it allows to implement of the BLE protocol whose capacity of transfer data is reduced to decrease the devices power consumption. Typically, the data transfer between UpNEA and the smartphone is performed continuously overnight, during recording: in this case, when the user stops the recording, then all data have already been transferred to the mobile device. Then, it takes 45 s for the cloud platform to process the data and write the results in the SQL database. Finally, the user can have access to its overnight data analysis after one minute from waking up. Summarizing the benefits of the proposed architectures: user comfort is boosted with respect to more cumbersome equipment employed for sleep monitoring (e.g., PSG), the signal quality maintained and the produced data size minimized.

#### 3.1.2. Breathing Rate

The PPG breathing rate estimation algorithm, integrated into the proposed sleep monitoring solution, was developed by Khreis in [14]. The implemented technique showed an absolute median error of 0.5 breath per minute (0.2–1.1 interquartile range 25th–75th) by segmenting the signal in windows of 32 s duration and 0.2 breath per minute (0.1–0.9) for the 64 s windowing. These results outperformed other methods, tested on the same database, and proposed by Pimentel in 2016 [77], Karlen in 2013 [20], Flemming in 2007 [78], Shelly in 2006 [79], and Nilson in 2000 [80].

#### 3.1.3. Apnea and Hypopnea

The algorithms for apnea and hypopnea detection, embedded in the novel system, were developed and published by the authors of [42]. Those methods showed an accuracy (Acc) of 75.1% for the detection of apneic/hypopneic events in one-minute segments, with 76.9% sensitivity (Se) and 73.2% specificity (Sp). In Table 5, the detection Se, Sp, and Acc values are reported for different types of sleep disordered breathing events: central apnea (CA) and central Hypopnea (CH), obstructive apnea (OA), and obstructive hypopnea (OH).

In addition, those algorithms were also tested on patients with a low AHI index (AHI ≤ 5), and their performances are presented in Table 6. To simplify, the following statement will clarify the table reading with an example: if we consider the central apnea detection, a sleep disruptive breathing event was considered FP when an apneic/hypopneic SDBE did not correspond to a central apnea annotation, even if other apnea types were present.

Comparing results of the apnea and hypopnea detector obtained on the overall database with those obtained for patients with low AHI, we see there is no substantial difference, confirming the robustness of the detector. Finally, those methods showed results comparable to those present in the literature and tested on the same database [81,82,83].

Additionally, in [42], a further classification was performed on the detected DAP events to discriminate between central and obstructive, apneic and hypopneic events. The classification was performed with a Fine Gaussian SVM classifier and showed 92.6% Acc in separating central from obstructive apnea, 83.7% in separating central apnea from central hypopnea, and 82.7% in separating obstructive apnea from obstructive hypopnea. The True Positive rate (TPr = Se) and False Positive (FP) rate (FPr = 1 − Sp) as well as the Acc and ROC area under the curve (AUC) are shown in Table 7. For comparison, on the same database, no other works were performed in classifying the SDBE subtypes.

Note that these results were obtained on signals acquired by the devices available at the UZ Leuven clinic and not directly with the proposed UpNEA device. However, the database signals were opportunely downsampled at 100 Hz, before being processed, to emulate the UpNEA acquisition. For costs and time limits, it was not possible to launch a clinical protocol for the presented device. Nevertheless, because the proposed platform transmits lossless data by its conception, the authors would conclude that it is possible to neglect the hardware validation.

### 3.2. Performances of the Novel Sleep Monitoring Platform

**Figure 7:**
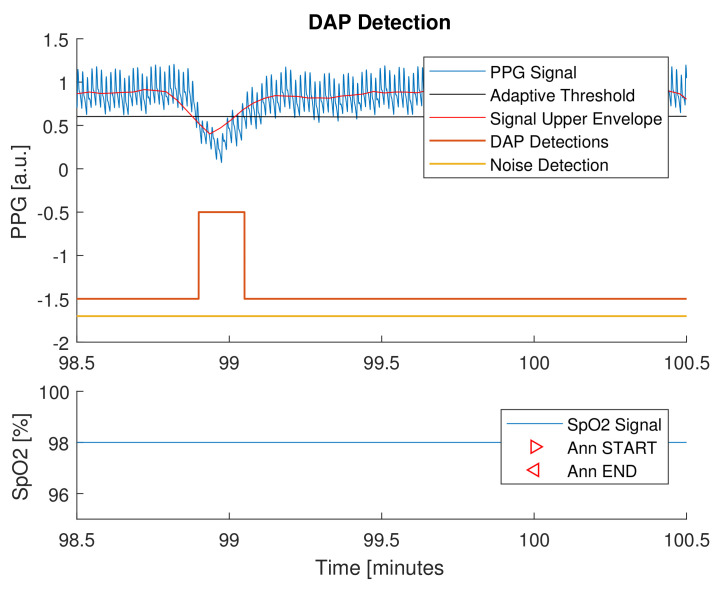
The DAP detection method is applied to a PPG signal.

Finally, the novel proposed platform has been employed to monitor four nights of sleep, on a 30-year-old healthy male subject not suspected to have apnea or hypopnea syndrome. The subject voluntarily wore the smart glove UpNEA for four consecutive nights and reported no sleep discomfort in wearing the acquisition device, as shown in Figure 1. As already described, the apnea and hypopnea detection analysis was conducted segmenting the whole night recordings in one-minute segments, implementing the algorithm in [42]. Despite the fact that even a completely healthy person could have some apneic/hypopneic episodes, for testing purposes, it was supposed that no apneas or hypopneas were present. The tests results showed a detection specificity of 96.2%; further details are represented in Table 8. An example of apnea and hypopnea detection is presented in Figure 7. The figure shows a time frame containing a DAP event but not a corresponding desaturation in the SpO${}_{2}$ signal: in that case, the DAP event was labeled as control. This example can explain the specificity value obtained on the four night recordings, where, most likely, a desaturation loss did not correspond to the majority of the detected DAP events. In conclusion, the authors propose these tests as support for a proof-of-concept of the novel sleep monitoring system.

## 4. Discussion

Is the authors wish that the presented sleep monitoring platform could inspire the translation of biomedical algorithms to new connected devices, passing by top-notch cloud computing solutions. The proposed system consists of a smart glove that is used to increase user comfort during sleep examination, with respect to the PSG golden standard procedure. In regard, as the four overnight recording trials revealed, the proposed equipment had poor sleep ease. Data from UpNEA are sent by a lossless compression protocol to a remote server. Furthermore, AeneA can be easily updated with new improved algorithms by redesigning the MATLAB${}^{\text{®}}$ deployed function and these changes could then be directly implemented in production. The proposed platform makes it easy and fast the transition from experimentation to production and allows the introduction of new functionalities, as the algorithms have been validated.

Concerning the implemented algorithms, the authors chose to implement in the platform those they already developed and published. In addition, the integrated AFib and PVC methods were chosen from a published study that embedded them in a wearable device worn by psychiatric patients. Each implemented algorithm presents its strengths and weaknesses.

The pulse detection implemented a prominence peak detector, tailored to the database used for validating the algorithms. A valid alternative would be to implement a Wiener filter like the one proposed in [84]. In consequence, the AFib and PVC detection methods would benefit from the integration of pulse rate variability information. We propose that the BR estimation would improve its performance if accelerometer data (already available for sleep stages) would have also been exploited for the task. The apnea and hypopnea detection and classification methods showed good results in highlighting SDBE and in classifying their nature. Nevertheless, a weak point is represented by their classification procedure that did not implement the LOSO strategy, as the detection did: for this purpose, further clinical trials will be required. Last but not least, the sleep stages classifier would benefit from the integration of PPG signal processing.

At this point, note that the strength of the presented system relies on the flexibility and scalability of cloud computing: flexibility is intended as the system capacity to introduce new functionalities and improve the implemented algorithms, without updating the acquisition devices; scalability, instead, allows the remote server (AWS EC2), to be easily upgraded (e.g., the number of users and threads can be augmented by only updating the AWS plan). This platform makes the transition from research to production easier and represents a fast way to remotely build new databases, for research purposes.

On the other hand, trials results obtained exploiting the platform in four overnight recordings showed high specificity values for apnea and hypopnea detection: those conclusions be correlated to the good health of the tested subject. Although these proof-of-concept outcomes have no clinical significance (because it was not possible to establish a comparison with PSG reference), the results are still indicative of the good health of the subject and encourage continued testing of the sleep monitoring system in a clinical context.

In comparison with similar products presented on the market, the authors’ research found two sleep monitoring systems that were revealed to be the most similar to the platform proposed in the present paper: ApneaBand and WatchPAt. Both systems implement PPG technology and consist, respectively, of a wrist band and a wrist band and a thimble. At the time of publication of the present paper, ApneaBand is not commercially available yet. Moreover, the smart band cannot acquire PPG finger signals (usually less noisy), but it exploits wrist PPG information to provide the overnight detection of apnea events. The algorithms are embedded in the device, and thereby they do not take the computational advantage of cloud computing. Results are presented to the user by physically connecting the device to a personal computer. In respect, WatchPAt is commercially available; takes as input PPG finger signals; and promises to detect central sleep apnea events along with heart rate, oximetry, actigraphy, body position, snoring, and chest motion information. As the presented sleep monitoring platform, WatchPAt, seems to integrate a cloud computing system with limited functionalities with respect to the proposed systems: it is not able to discriminate between central and obstructive apnea and hypopnea, nor detect AFib and PVC.

## 5. Conclusions

The proposed sleep monitoring platform represents a proof-of-concept for a non-invasive sleep monitoring system. The zero data loss encoding method embedded in its hardware allows the system to communicate to UpNEA devices without corrupting signals quality and compressing the transmitted information. All algorithms integrated into the platform were tested in different studies, already published in the literature. In particular, the authors choose to embed the breathing rate estimation algorithm proposed by Khreis et al. that outperformed other methods tested on the CAPNOBASE database. As regards the apnea and hypopnea detection and classification methods, the authors exploited their own work that exhibited good performances on clinical trials and was published in a separate paper. The point of strength of this platform consists of its flexibility and scalability, and targets individuals but also clinics that practice polysomnography. It will be of great interest to continue validating the proposed sleep monitoring platform as a medical solution complementary to polysomnography.

## Figures and Tables

**Table 1:** List of data descriptors.

Data Descriptor	Data Type	[bits]
0	*DD*: PPG Delta Difference	8
10	*GB*: PPG Garbage	0
110	*AV*: PPG Absolute Value	18
1110	*SF*: PPG Scale Factor	3
11110	SpO${}_{2}$	6
111110	Movements	16

**Table 2:** Encoding data block row example.

110	AV value	0	DD value	0	DD value	1110	SF value	DD value	0	DD value	10	110	AV value

**Table 3:** Total number of sleep disordered breathing events, per category, in the database.

	CA	CH	OA	OH	MA
Number of events	765	689	4984	14,140	750
Percentage of events	3.6%	3.2%	23.4%	66.3%	3.5%

**Table 4:** SQL tables data fields.

*NightSleepSummary* SQL table fields
UserId, UpNEAId, EventStartDate, EventEndDate, LightSleepDuration, DeepSleepDuration, SleepDuration, WakeUpsCounter, ApneaCounter, MeanHeartRate, MeanBreathingRate, MeanOxygenSaturation, PrematureVentricularContractionCounter, AtrialFibrillationCounter, BradycardiaCounter, TachycardiaCounter.
***SleepEvents*** **SQL table fields**
UserId, EventStartDate, HeartRate, BreathingRate, OxygenSaturation, IsApnea, ApneaType, IsPrematureVentricularContraction, IsAtrialFibrillation, IsBradycardia, IsTachycardia.

**Table 5:** Apnea and hypopnea detection results.

	CA	CH	OA	OH
Se [%]	86.6	73.3	86.4	76.2
Sp [%]	55.3	57.4	57.2	68.2
Acc [%]	70.9	65.4	71.8	72.2

**Table 6:** Apnea/Hypopnea Detection results on patients with AHI ≤ 5.

Event Type	Se [%]	Sp [%]	Acc [%]
All	79.9	75.8	76.1
Central Apnea	100	72.0	72.2
Central Hypopnea	83.6	72.4	72.6
Obstructive Apnea	88.9	63.6	63.8
Obstructive Hypopnea	76.8	74.2	74.3

**Table 7:** Respiratory events classification performances for Fine Gaussian SVM.

		TPr [%]	FPr [%]	Acc [%]	AUC
C-O	C	95	10	93	0.97
	O	90	5		
CA-CH	CA	86	19	84	0.91
	CH	82	14		
OA-OHA	OA	85	20	83	0.89
	OHA	81	14		

**Table 8:** Apnea and hypopnea detection applied on a healthy subject.

Night	1	2	3	4	Total
Sp [%]	95.4	97.9	92.1	99.3	96.2

## Data Availability

The PPG breathing rate estimation algorithm was validated on the public CAPNOBASE database. The algorithms for apnea and hypopnea detection and classification were tested on 96 overnight signals, recorded at the UZ Leuven hospital, from patients with sleep apnea/hypopnea syndrome and without any kind of collateral morbidity. The novel proposed platform has been employed to monitor four nights of sleep, on a 30-year-old healthy male subject not suspected to have apnea or hypopnea syndrome.
